# Lacrimal Secretory Niche Remodeling in Dry Eye Disease: Single-Cell and Spatial Insights into Acinar Dysfunction

**DOI:** 10.3390/cells15191828

**Published:** 2026-10-08

**Authors:** Yuan Zhong, Xiyuan Liu, Qinghua Peng, Jun Peng

**Affiliations:** 1 Faculty of Traditional Chinese Medicine, Hunan University of Chinese Medicine, Changsha 410208, China; zhongyuan@stu.hnucm.edu.cn (Y.Z.); liuxiyuan@stu.hnucm.edu.cn (X.L.); pengqinghua@hnucm.edu.cn (Q.P.); 2 Institute of Ophthalmology and Otolaryngology of Chinese Medicine, Changsha 410208, China

**Keywords:** dry eye disease, lacrimal gland, acinar cell, acinar secretory competence, lacrimal secretory niche, single-cell RNA sequencing, spatial transcriptomics, functional restoration

## Abstract

**Highlights:**

**What are the main findings?**

- Lacrimal hyposecretion reflects changes in neural regulation, epithelial secretion, and tissue support; reduced tear output alone does not identify the underlying lesion.
- Single-cell and spatial studies identify candidate mechanisms whose functional significance requires testing at the cellular, glandular, or lacrimal functional-unit level.

**What are the implications of the main findings?**

- Residual functional reserve describes potentially recruitable secretion from surviving tissue and its supporting niche; it remains a working concept rather than a validated clinical measure.
- Recovery studies should match their endpoints to the proposed mechanism and distinguish improved secretion from tissue regeneration and sustained ocular-surface benefit.

**Abstract:**

Reduced tear output can arise from altered neural regulation, impaired epithelial secretion, or loss of secretory tissue, with different implications for recovery. This focused narrative review integrates physiological, single-cell, spatial, organoid, and mechanistic studies identified through a structured PubMed search and targeted reference checking. We examine how remodeling of the lacrimal secretory niche affects acinar secretory competence, the capacity of viable acinar cells to convert stimulation into coordinated fluid, electrolyte, and protein secretion. Omics studies identify disease-associated expression patterns and candidate interactions, while selected perturbation studies link neural, inflammatory, and metabolic mechanisms to secretory dysfunction. We use residual functional reserve as a working concept for secretion that surviving tissue and its supporting niche can recruit through stimulation or relief of suppression. Potentially reversible functional suppression, persistent niche dysfunction, and structural failure describe overlapping constraints on secretion, not validated disease stages. These descriptions are distinct from the cellular, glandular, and lacrimal functional-unit levels at which outcomes are measured. The framework organizes questions about mechanism and recovery but does not establish a clinical treatment algorithm. Progress requires functional testing of candidate mechanisms and assessment of recovery at the level relevant to each claim.

## 1. Introduction

Dry eye disease (DED) is a heterogeneous disorder of tear-film and ocular-surface homeostasis [1]. In experimental models, disruption of lacrimal innervation or corneal sensory input reduces tear secretion [2,3]. Conversely, blocking excessive sympathetic signaling improves secretion in several mouse models of dry eye [4]. Structural injury provides another route to hyposecretion: prolonged lacrimal duct obstruction causes acinar damage and limits recovery after release in rats [5]. Reduced tear output therefore identifies a functional deficit but does not, by itself, distinguish altered neural regulation from damage to secretory tissue. Interpreting the underlying lesion and its potential reversibility requires complementary evidence on tissue integrity and secretory responsiveness.

Lacrimal secretion depends on a spatially organized, multicellular tissue system [6,7]. Polarized acinar cells generate primary lacrimal fluid, while ducts, myoepithelial cells, nerves, vessels, stromal cells, and immune populations provide transport, regulation, and local support [7]. We refer to this integrated system as the lacrimal secretory niche. Within this niche, acinar secretory competence denotes the capacity of viable acinar cells to convert stimulation into coordinated fluid, electrolyte, and protein secretion. This cell-level capacity provides a common reference for examining how epithelial defects and changes in the surrounding tissue contribute to hyposecretion.

Single-cell and spatial studies resolve lacrimal cell populations, disease-associated transcriptional programs, and the tissue distribution of inflammatory changes [8,9,10,11,12]. These approaches can identify candidate acinar states and interactions that would be obscured in whole-tissue measurements. However, transcriptional differences and spatial proximity do not establish whether a candidate mechanism impairs secretion or whether the affected tissue can recover [13,14]. The unresolved task is to connect these molecular and spatial observations with direct measurements of secretion and the effects of targeted perturbation.

This review integrates physiological, omics, and mechanistic evidence to examine how remodeling of the lacrimal secretory niche impairs acinar function. We use residual functional reserve as a working concept for the secretory capacity that surviving tissue and its supporting niche retain. This capacity may be recruited by appropriate stimulation or by relieving suppressive influences. Section 5.5 uses this concept to discuss potentially reversible functional suppression, persistent niche dysfunction, and structural failure as overlapping descriptions, rather than validated disease stages. Separately, we distinguish acinar secretory competence, intact-gland secretory output, and lacrimal functional-unit performance as levels of functional assessment. These levels do not correspond one-to-one to the three descriptions of dysfunction. Omics findings generate candidate explanations, while perturbation and functional measurements test their relevance at the appropriate level.

## 2. Literature Search and Evidence Selection

A structured PubMed search was conducted to support this focused narrative review. The database was searched for articles published between 1 January 2010 and 26 August 2026. Five deliberately overlapping search blocks covered: (i) single-cell, single-nucleus, spatial-transcriptomic, transcriptomic, and organoid studies; (ii) neural, neuroendocrine, stress-related, and circadian regulation; (iii) immune remodeling and epithelial–immune communication; (iv) acinar secretion, metabolism, mitochondrial and organelle stress, autophagy, and oxidative-stress-related processes; and (v) tissue repair, regeneration, therapeutic intervention, and biomarkers. Each block combined lacrimal gland terms with the corresponding domain-specific terms.

The five searches returned 133, 285, 1869, 895, and 840 records, respectively. Because the search blocks intentionally overlapped, the 4022 retrieved records were merged and deduplicated by PubMed identifier (PMID). This process left 2453 unique records for title and abstract screening. Original studies were eligible for the focused evidence pool when they directly examined human or animal lacrimal gland tissue, lacrimal gland-derived cells, organoids, or multicellular models. Eligible studies contributed evidence on cellular composition, spatial organization, acinar secretory competence, disease-associated remodeling, or functional restoration. Case reports, commentaries, and reports without a direct contribution to the lacrimal secretory niche were not used as primary evidence. Methodological and data-resource articles were retained only when they provided a distinct biological or analytical contribution.

Title and abstract screening excluded 2043 records, leaving 410 articles for detailed relevance assessment. Eligibility and evidence roles were determined from the title, abstract, and, where required, the full report. Full reports were prioritized for studies supporting the principal single-cell, spatial, and mechanistic claims of this review. Studies were assessed according to their direct relevance to lacrimal gland biology, study design, cellular or spatial resolution, functional or perturbational evidence, and non-redundancy within each review theme. This process yielded a focused evidence pool of 154 studies. The remaining 256 articles were not used in the core synthesis because they provided only background or methodological information, lacked sufficient specificity, duplicated more informative evidence, or fell outside the secretory-niche focus. Earlier foundational studies and additional contextual references were identified through targeted reference checking and were considered separately from the database-derived record counts.

Evidence was interpreted according to the relationship tested. Descriptive omics findings, pathway enrichment, ligand–receptor inference, and spatial colocalization were treated as associative or hypothesis-generating evidence. Tissue imaging and protein measurements strengthened cell-state and spatial assignments but did not, by themselves, establish causality. Perturbation studies supported mechanistic interpretation when a defined intervention was linked to the relevant molecular, cellular, or tissue response. Claims about secretory recovery additionally required measurements of acinar secretion or intact-gland output. Cell-specific perturbation, target engagement, and complementary rescue experiments strengthened attribution of an effect to the proposed mechanism. The evidence map is qualitative and is not a formal evidence-grading or risk-of-bias instrument. The PubMed-only scope and narrative selection process limit the completeness of this synthesis.

## 3. Physiological Basis of Acinar Secretory Competence

Acinar secretory competence describes the capacity of viable epithelial cells to respond to stimulation and secrete fluid, electrolytes, and proteins. Intact-gland output also depends on neural input, blood supply, myoepithelial activity, ducts, and tissue organization [2,3,4,6,15]. At the level of the lacrimal functional unit, tear delivery and ocular-surface effects must also be considered [1,6,15]. The following sections begin with the acinar cell and then examine the tissue systems that support its activity.

### 3.1. Acinar Cell Architecture and Secretory Cargo

Lacrimal acinar cells are organized to produce and release secretory cargo. Rough endoplasmic reticulum (ER) supports protein synthesis, and the Golgi apparatus processes and sorts newly synthesized proteins. Secretory granules accumulate toward the apical pole, close to the acinar lumen. Mitochondria supply energy, while the endoplasmic reticulum also stores Ca^2+^ for stimulus-dependent signaling [6,15,16,17]. This arrangement places biosynthesis, cargo storage, and release within a polarized epithelial cell.

Polarity gives secretion its direction. The apical membrane faces the lumen, whereas the basolateral surface faces the interstitium and receives neural and circulating signals. Junctions between neighboring cells maintain the epithelial barrier and separate these membrane domains. Ion channels, transporters, and vesicle-fusion machinery occupy specific membrane locations, allowing the cell to deliver fluid and proteins into the lumen [15,18].

Proteins reach the lumen by more than one route. Regulated secretory granules store cargo for stimulus-dependent exocytosis, whereas constitutive secretion provides a separate route for protein release [17]. Transcytosis transports material taken up at the basolateral surface to the apical membrane. For example, the polymeric immunoglobulin receptor (pIgR) carries dimeric immunoglobulin A (IgA) across acinar cells, linking epithelial transport to mucosal defense. Experiments in rabbit lacrimal acinar cells distinguish this transcytotic route from a pIgR-containing regulated secretory pathway [19]. These routes share an apical destination but differ in cargo handling and control. This organization explains how cargo reaches the lumen; the stimulus-dependent control of release is considered next.

### 3.2. From Receptor Activation to Fluid and Protein Secretion

Acetylcholine and vasoactive intestinal peptide (VIP) stimulate lacrimal secretion through parasympathetic signaling; noradrenaline provides context-dependent sympathetic regulation [4,15]. Their receptors activate intracellular pathways that control ion transport and protein release. The cellular steps are considered here, while the neural circuits that coordinate glandular activity are discussed in Section 3.4.

Activation of muscarinic acetylcholine receptor M3 (M3) stimulates phospholipase C and the production of inositol 1,4,5-trisphosphate, which releases Ca^2+^ from the endoplasmic reticulum. Depletion of these stores promotes store-operated Ca^2+^ entry and sustains the secretory signal. Cytosolic Ca^2+^ activates ion channels and supports granule exocytosis [15,16]. VIP acts mainly through vasoactive intestinal peptide receptors (VPAC receptors) and cyclic adenosine monophosphate (cAMP)-dependent signaling [15]. These pathways regulate overlapping components of secretion, but their effects on fluid and protein output need not be identical.

Fluid secretion follows the movement of ions across the acinar epithelium. Na^+^/K^+^-dependent adenosine triphosphatase (Na^+^/K^+^-ATPase) and coupled transport processes maintain the gradients needed for Cl^−^ accumulation and release into the lumen. Na^+^ and water then follow electrochemical and osmotic gradients [15,18,20]. Both transcellular and paracellular pathways contribute to water movement, with aquaporins facilitating transport across cell membranes [20]. Thus, effective fluid secretion requires coordinated transport and preserved epithelial polarity.

The location of a transporter matters as well as its abundance. In isolated mouse lacrimal acinar cells, electrophysiology localized Ca^2+^-activated K^+^ conductance predominantly to the apical membrane, alongside Cl^−^ conductance. Immunostaining also detected apical Na^+^/K^+^-ATPase [18]. These observations led to a proposed contribution of apical pumps to K^+^ reabsorption and primary fluid composition. The localization data support this model, but they do not by themselves quantify the contribution of each transport route to intact-gland secretion.

Protein release requires granules to reach, dock at, and fuse with the apical membrane. Rab-family guanosine triphosphatases (GTPases), cytoskeletal motors, and soluble N-ethylmaleimide-sensitive factor attachment protein receptor (SNARE) proteins regulate these steps [17]. Ca^2+^ and cAMP signaling couple stimulation to this machinery, while the amount and composition of releasable cargo depend on the biosynthetic and trafficking processes described above. Because fluid transport and protein exocytosis use partly distinct machinery, a change in one output cannot be assumed to represent the other.

### 3.3. Multicellular and Spatial Coordination of Acinar Secretion

Acinar secretion operates within a multicellular tissue architecture. Ducts receive primary acinar fluid and modify its water and electrolyte composition. Lacrimal duct organoids retain polarized expression of cystic fibrosis transmembrane conductance regulator (CFTR), Na^+^/K^+^/2Cl^−^ cotransporter 1 (NKCC1), and aquaporin 4 (AQP4) and exhibit secretagogue-responsive fluid movement [21,22]. These findings establish active transport by ductal epithelium. However, the quantitative contribution of ducts to final lacrimal fluid composition and intact-gland output remains unresolved in vivo [21,22].

Myoepithelial cells surround acini and proximal ducts and contract in response to physiological stimulation. Oxytocin-induced myoepithelial contraction has been associated with increased tear output in mice [23,24]. Nevertheless, myoepithelial contraction and intact-gland secretory output represent different functional endpoints. Changes in tear output also depend on acinar secretion, ductal patency, neural input, and ocular-surface collection. Proposed roles in epithelial polarity, basement-membrane organization, and paracrine signaling require further functional validation [7,23,24].

Neural and vascular structures are closely integrated with the epithelial–myoepithelial unit. Autonomic fibers approach acinar and myoepithelial cells, providing an anatomical basis for coordinated activation of neighboring secretory units [2,3,15]. The vascular network supplies the water, electrolytes, oxygen, and metabolic substrates required for secretion [6,15]. These relationships connect epithelial execution to neural drive and metabolic support.

Fibroblasts, endothelial cells, pericytes, and resident immune populations also shape the local environment. Transcriptional and spatial studies associate these populations with extracellular-matrix maintenance, vascular stability, trophic support, and immune surveillance [7,8,9,12]. Their proposed roles derive largely from expression profiles, localization, and predicted intercellular communication.

The spatial arrangement of acini, ducts, nerves, vessels, and stromal cells provides the anatomical context for secretion [7,8,9,12]. Ductal transport and myoepithelial contraction have direct experimental support [21,22,23,24], whereas many stromal and immune interactions remain candidates identified from tissue organization. This distinction separates demonstrated supporting functions from interactions suggested by proximity.

### 3.4. Neural, Neuroendocrine, and Circadian Modulation of the Secretory State

Neural, neuroendocrine, and circadian signals coordinate secretion with ocular-surface demand and physiological timing. Sensory nerves in the cornea and conjunctiva detect cooling, evaporation, mechanical stimulation, and irritation [25]. These signals are integrated centrally and relayed to the lacrimal gland through autonomic pathways [3,15,25]. The resulting output regulates epithelial secretion, vascular tone, and myoepithelial activity [15,23,24].

Parasympathetic signaling provides the principal acute secretory drive through cholinergic and peptidergic pathways [15]. Sympathetic signaling provides additional, context-dependent modulation through adrenergic receptors [4,15]. Autonomic innervation also contributes to long-term glandular maintenance. Experimental denervation alters tear secretion, gene expression, and tissue organization [2].

Peptide and hormonal signals influence lacrimal gland activity through different routes. VIP regulates epithelial secretion, whereas oxytocin can stimulate myoepithelial contraction [15,23,24]. Androgen and estrogen receptor expression has been detected in human lacrimal tissue, but receptor expression alone does not establish a secretory response [26]. In stressed mice, β-adrenergic blockade and inhibition of glucocorticoid synthesis modify glandular abnormalities and tear secretion [27]. These findings support endocrine and neuroendocrine regulation without assigning all effects to one epithelial pathway.

Lacrimal secretion also varies across the daily cycle. Wild-type mice show rhythmic tear secretion and lacrimal clock-gene expression, whereas these rhythms are disrupted in Cry1/Cry2-deficient mice [28]. Chronic stress alters rhythmic immune, metabolic, and neural expression programs in the gland [27]. These observations link circadian regulation to secretion, but do not establish how each downstream expression change contributes to reduced output.

**Figure 1:**
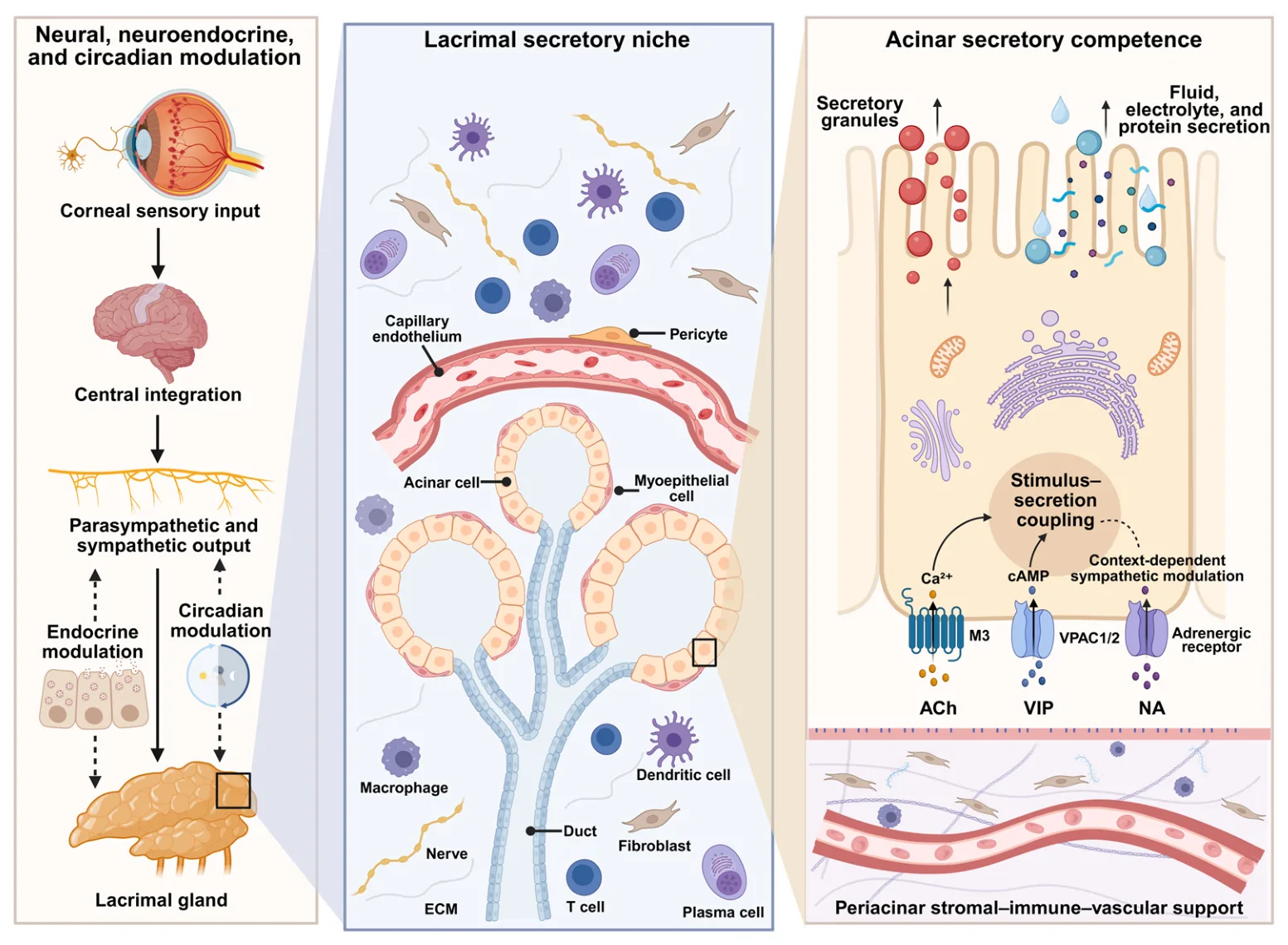
Physiological organization of the lacrimal secretory niche and acinar secretion. Left, corneal sensory input is integrated centrally and relayed to the gland through autonomic pathways. Endocrine and circadian influences provide additional regulation. Center, acini and ducts are shown with myoepithelial, neural, vascular, stromal, and resident immune components. Their positions illustrate tissue organization rather than measured cell proportions or verified signaling between every neighboring cell type. Right, the polarized acinar cell links receptor activation to intracellular signaling and luminal secretion. ACh–M3 signaling acts mainly through Ca^2+^, while VIP–VPAC signaling acts mainly through cAMP; NA provides context-dependent adrenergic modulation. The endoplasmic reticulum, Golgi apparatus, mitochondria, and secretory granules support cargo production, processing, and release. Fluid transport and protein exocytosis are related but distinct outputs. Arrows summarize physiological relationships; dashed arrows indicate modulation, without specifying its magnitude or direction in every condition. This schematic corresponds to the structural sequence in Section 3.1 and the stimulus-to-output sequence in Section 3.2. ACh, acetylcholine; cAMP, cyclic adenosine monophosphate; ECM, extracellular matrix; M3, muscarinic acetylcholine receptor M3; NA, noradrenaline; VIP, vasoactive intestinal peptide; VPAC, vasoactive intestinal peptide receptor; VPAC1/2, vasoactive intestinal peptide receptors 1 and 2.

Neural, neuroendocrine, and circadian inputs regulate acinar secretion across acute and sustained timescales [15,27,28]. Together, they connect ocular-surface demand with epithelial execution, metabolic support, and tissue maintenance. Figure 1 summarizes the physiological organization of this regulatory system.

## 4. Disease-Associated Remodeling Revealed by Single-Cell and Spatial Omics

### 4.1. Cellular Heterogeneity of the Normal Lacrimal Gland

Human and mouse single-cell atlases consistently identify the major cellular compartments of the lacrimal gland. These include acinar, ductal, myoepithelial, stromal, vascular, and immune populations. Each compartment contains additional transcriptional heterogeneity. Together, these datasets provide the normal cellular reference required to interpret disease-associated changes in epithelial state and niche composition [7,9,12].

Single-cell profiling of adult human lacrimal tissue has resolved major epithelial and non-epithelial populations and enabled comparison with cultured organoids [12]. In this study, expandable human and mouse organoids retained duct-associated features. Human organoids responded to neurotransmitters and produced mature tear products after transplantation into mice. Native tissue and organoid datasets therefore provide complementary information, but culture composition should not be assumed to reproduce the intact gland.

Mouse atlases provide greater sampling depth and reveal additional epithelial heterogeneity. Reported populations include conventional acinar cells, Tfrc-high and Car6-high acinar states, Car6-positive cells near the acinar–ductal junction, and several ductal populations. The number and boundaries of these subclusters vary with strain, sex, tissue sampling, sequencing depth, and annotation strategy. Major cellular compartments are reproducible across datasets. By contrast, finer epithelial states require repeated detection, anatomical localization, and functional characterization before they can be regarded as stable biological populations [7,9].

Sex is an important source of physiological variation in the mouse lacrimal gland. Acinar cells show sex-biased expression of lipocalins, secretoglobins, and other secretory products. These differences affect baseline comparisons and the interpretation of disease-associated changes. Human evidence for sex-related acinar programs remains limited. Comparative studies should therefore account for sex and hormonal context in both experimental design and data interpretation [7].

Healthy lacrimal glands also contain diverse resident immune populations, including macrophages, dendritic cells, T cells, B cells, plasma cells, innate lymphoid cells, and mast cells. Stromal and vascular compartments show comparable heterogeneity. Disease-associated immune remodeling can involve changes in abundance, activation state, or spatial distribution among populations already present in healthy tissue. Normal reference datasets are therefore necessary to distinguish altered cellular states from the emergence of genuinely new populations [7,9].

Several epithelial populations have been proposed as progenitor or plastic states. These include Krt14-positive basal ductal cells, Aldh1a1-positive ductal cells, and Sox10-positive epithelial populations. Lineage-tracing studies support multilineage contributions from adult Sox10-expressing cells. The roles of other candidate populations in routine maintenance and chronic injury remain unresolved. Moreover, differentiated epithelial cells can express keratins or developmental regulators during stress and repair. Marker expression alone is therefore insufficient for progenitor assignment, which requires lineage tracing, spatial localization, and functional differentiation [7].

Collectively, normal lacrimal gland atlases define a tissue with substantial cellular and transcriptional heterogeneity. This baseline variation reflects cellular identity, physiological state, sex, and experimental context. Disease-associated remodeling must be interpreted against each of these sources of normal variation.

### 4.2. Acinar Cell-State Remodeling in Lacrimal Dysfunction

Disease-focused datasets report changes in secretory gene expression, inflammatory and stress-response programs, metabolic pathway signatures, and epithelial composition [9,10,11]. The affected populations and expression patterns differ across models. These studies therefore identify several forms of acinar remodeling, without yet defining a universal dysfunctional cell state.

Genetic models of Sjögren-like disease illustrate this context dependence. MRL/lpr and NOD.H2b glands share broad interferon-responsive programs but differ markedly in immune composition. T-cell infiltration predominates in MRL/lpr glands, whereas NOD.H2b glands contain mixed B- and T-cell infiltrates. Their parenchymal responses also differ. Thus, interferon activation represents a recurrent disease axis embedded within model-specific epithelial and immune environments [9].

Scopolamine combined with desiccating stress induces acute lacrimal dysfunction. Single-nucleus profiling identified reduced acinar representation and weaker mature secretory programs, together with increased ductal and myoepithelial representation and enhanced immune signaling. Acinar cells nevertheless remained a major epithelial population, indicating substantial state remodeling within retained glandular tissue. Relative cell fractions require compositional interpretation because expansion or differential recovery of other populations can lower the apparent acinar proportion. Histological quantification and stimulated secretion provide complementary measures of acinar preservation and intact-gland secretory output [10,13].

Single-cell profiling of a mouse model of chronic lacrimal graft-versus-host disease (GVHD) found lower relative representation of acinar, myoepithelial, and endothelial cells, together with increased T-cell and macrophage representation. Myoepithelial cells showed reduced expression of genes associated with secretion and extracellular-matrix synthesis. Fibroblasts had inflammatory expression profiles, while endothelial changes involved immune recruitment and cell junctions. An Lrg1-high epithelial population was also reported [11]. Its origin and persistence cannot be determined from a single sampled disease stage.

Jin et al. compared murine lacrimal glands at acute and chronic GVHD stages and reported subtype-specific differences in glycan- and lipid-metabolism signatures [29]. Their analyses also predicted changes in fibroblast–T-cell and myeloid–epithelial communication. Immunofluorescence supported the presence of infiltrating plasmacytoid dendritic cells and mast cells, but did not establish the activity of the predicted ligand–receptor pairs. These are comparisons between sampled stages rather than longitudinal observations of individual acinar cells.

Whether these metabolic signatures reflect adaptation, impaired function, or both remains unresolved. We consider reduced biosynthetic demand as one possible response to stress, and loss of metabolic flexibility as a possible constraint on sustained secretion. These are interpretations of stage-associated expression patterns, not measured metabolic outcomes [29]. Testing a sequence from adaptation to failure would require measurements of metabolic activity and secretion, together with interventions directed at the proposed mechanism.

In aging glands, integrated bulk and single-cell analyses associated Plin2 expression with immune and metabolic signatures. Plin2 was reduced in fibroblast and monocyte/macrophage/dendritic-cell populations in the analyzed datasets [30]. Expression analysis and computational feature selection nominated Plin2 as a candidate regulator; its effect on lacrimal secretion remains untested.

The recurring finding across these datasets is altered acinar expression in model-specific tissue settings. Terms such as stress-adapted, transitional, or selectively surviving describe hypotheses about those changes. Survival advantage, lineage transition, and retained secretory capacity require temporal or lineage evidence and appropriate functional measurements [9,10,11,13,14,29,30].

### 4.3. Spatial Reorganization of Immune and Stromal Niches

Spatial studies place epithelial changes within the surrounding inflammatory and stromal tissue. In affected glands, immune-rich regions and epithelial regions show different expression profiles, providing a way to examine local rather than whole-gland associations [8]. These maps identify where candidate processes occur, while leaving their direction and functional consequences to be tested.

In NOD.H2b glands, Visium profiling associated immune-rich regions with leukocyte- and macrophage-related expression, including a Tyrobp-centered network. Epithelial regions showed reduced expression of genes involved in mitochondrial, amino-acid, and lipid-metabolic pathways. Inflammatory foci also varied with age and sex [8]. Because each spot samples multiple cells, these results support regional associations rather than direct communication between particular cell pairs.

In thrombospondin-1-deficient glands, digital spatial profiling identified acinar stress and secretory signatures, ductal mitochondrial and immune-related signatures, and myoepithelial contractile and matrix-related signatures. Reduced acinar Pigr expression was accompanied by lower secretory IgA in tears [31]. The tear measurement provides a functional correlate of the transcriptional finding, although it does not establish that reduced Pigr caused the change.

The same study identified immune infiltrates around ducts, with antigen-presenting-cell, T follicular helper-cell, and germinal-center B-cell signatures. Epithelial major histocompatibility complex class II (MHC II) expression and adjacent immune cells positive for C-X-C motif chemokine ligand 13 (CXCL13) provided additional evidence of a periductal inflammatory niche [31]. These observations locate the epithelial and immune abnormalities within a periductal niche.

Ligand–receptor analyses nominate fibroblast–immune, myeloid–epithelial, and lymphocyte–epithelial interactions across models [8,11,29,31]. Spatial proximity can make such predictions anatomically plausible, but proximity alone does not demonstrate ligand delivery or receptor activation. Protein localization and downstream signaling measurements provide further support; perturbing the proposed sender, receiver, or signaling pathway is needed to test its contribution to dysfunction [14].

Stronger causal evidence is available in the Aire-deficient mouse model. Single-cell ribonucleic acid (RNA) and T-cell receptor sequencing identified clonally expanded memory T cells positive for C-C motif chemokine ligand 5 (CCL5) and cluster of differentiation 8 (CD8) near acini. CD8^+^ T-cell depletion or interferon gamma (IFNγ) deficiency reduced inflammatory infiltration and acinar atrophy [32]. These interventions implicate CD8^+^ T cells and IFNγ in tissue injury in this model; their relevance to other forms of human Sjögren disease remains to be tested.

Together, these studies show why spatial context matters: an acinar expression profile can sit beside an immune aggregate, a ductal infiltrate, or altered stroma [8,31,32]. The location helps identify which interactions to test. It should not be used on its own to assign causal direction or predict recovery.

### 4.4. Interpretive Considerations for Current Omics Evidence

Interpreting lacrimal gland omics requires separating biological differences from effects of sampling and measurement. Large, fragile acinar cells may be recovered less efficiently during dissociation, and released secretory transcripts can contribute ambient RNA. Single-nucleus sequencing can reduce some cell-recovery biases, but samples nuclear rather than whole-cell RNA and therefore detects secretory transcripts differently [7,9,10,13]. Apparent differences in cell abundance or secretory programs need to be interpreted with these platform effects in mind.

Cell proportions in single-cell datasets are inherently compositional. Expansion of immune or stromal populations can lower the relative acinar fraction without a proportional reduction in absolute acinar cell number. Histological quantification, stereology, or lineage-based approaches are needed when absolute cellular depletion is inferred. Sequencing depth, analytical resolution, marker selection, batch correction, and sample composition also influence the definition of finer epithelial states [10,13].

Biological sampling introduces additional variation. Sex, age, circadian phase, disease duration, gland type, and tissue-processing conditions affect both cellular composition and transcriptional state. Pooling glands increases cell yield but removes information on inter-animal variability. These variables require particular attention when studies compare different disease models or sampling stages [7,9,10,11,13,28,30].

Cross-model comparisons must preserve etiological context. Pharmacological hyposecretion, autoimmune dacryoadenitis, GVHD, neural injury, diabetes, and aging impose different combinations of neural, immune, metabolic, and structural stress. Recurrent transcriptional programs can indicate convergent responses, whereas model-restricted states can reflect disease-specific mechanisms. Most disease datasets currently derive from mouse lacrimal glands. Human single-cell evidence remains concentrated in healthy tissue and organoid systems [8,9,10,11,12,29,30,31,32].

Spatial methods provide anatomical information at different levels of resolution. Visium captures regional transcriptional patterns from multicellular spots. Digital spatial profiling depends on region selection, segmentation, and deconvolution. Ligand–receptor analysis, pathway enrichment, and pseudotime identify candidate interactions or state relationships rather than demonstrated mechanisms. Support from cellular localization, protein-level measurements, downstream pathway activity, and targeted perturbation increases their interpretive strength [8,14,31,33].

Transcript abundance represents only one layer of secretory function. Receptor localization, protein phosphorylation, organelle activity, metabolic flux, ion transport, and vesicle fusion can change without parallel alterations in messenger RNA (mRNA) abundance. Omics-defined acinar states acquire functional significance when linked to direct measurements of epithelial responsiveness, acinar secretory competence, or intact-gland secretory output [13,14].

**Figure 2:**
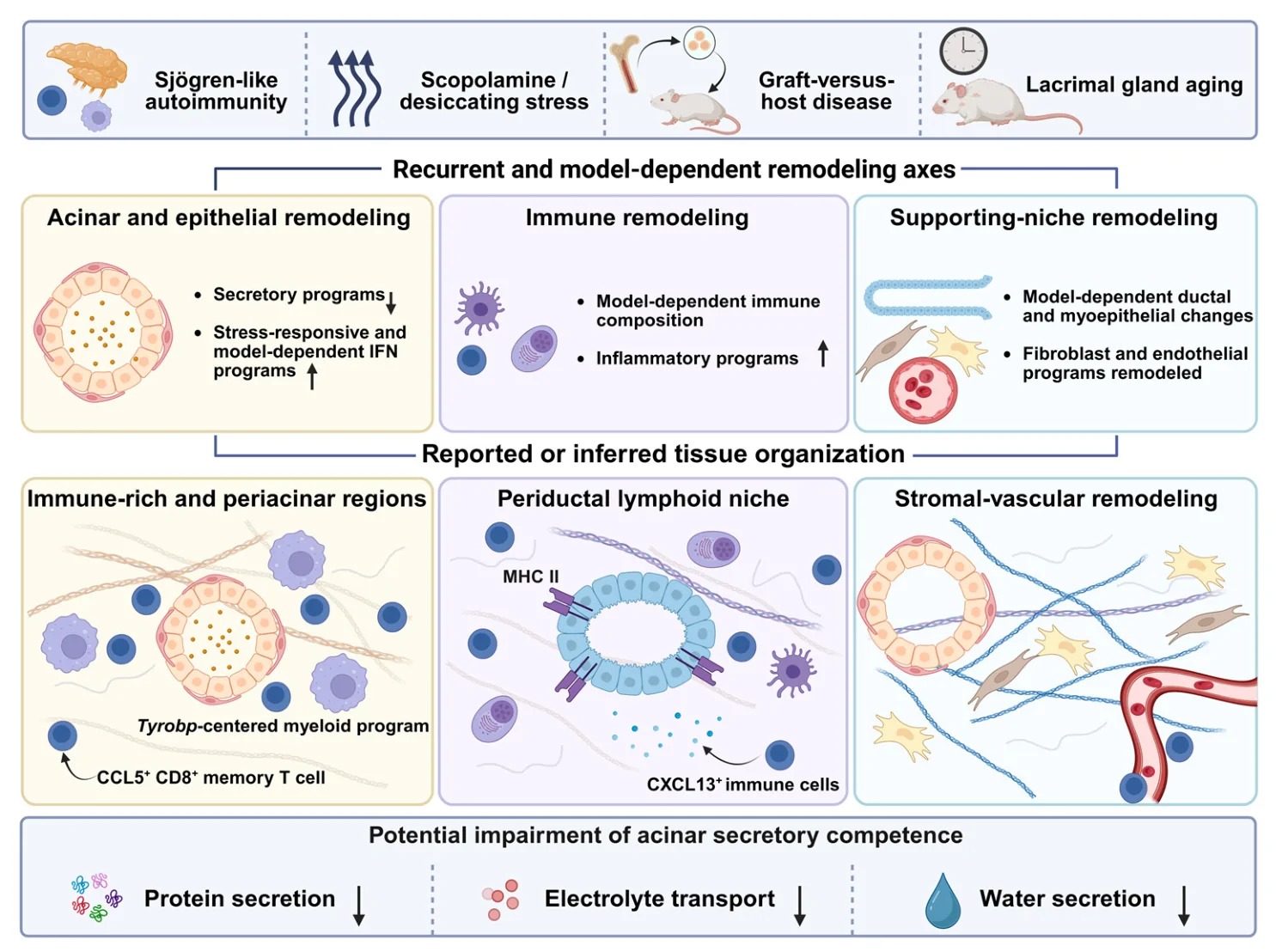
Expression patterns and spatial organization reported across lacrimal disease models. The top row identifies model contexts, and the middle row summarizes epithelial, immune, and supporting-cell expression changes. Upward and downward arrows describe reported expression trends, not measured secretion, metabolic flux, or a common response in every model. The lower tissue panels combine findings from separate studies rather than depict one experimentally mapped gland. CCL5^+^ CD8^+^ memory T cells were localized near acini in Aire-deficient mice [32]. The Tyrobp-centered myeloid program was associated with immune-rich regions in NOD.H2b glands [8]. In thrombospondin-1-deficient glands, spatial profiling and imaging identified periductal immune organization; ductal cultures provided additional evidence of inflammatory responses [31]. The MHC II and CXCL13 labels summarize these related observations and do not establish antigen transfer or a causal recruitment pathway. Stromal and vascular drawings summarize expression and anatomical findings rather than validated cell-to-cell signaling. The bottom row presents possible secretory consequences that require functional testing. It does not report three measured reductions or assign the proposed dysfunction states in Section 5.5. CCL5, C-C motif chemokine ligand 5; CD8, cluster of differentiation 8; CXCL13, C-X-C motif chemokine ligand 13; GVHD, graft-versus-host disease; IFN, interferon; MHC II, major histocompatibility complex class II; Tyrobp, TYRO protein tyrosine kinase-binding protein.

Comparisons are most informative when differences in disease model, sampling, and measurement are considered alongside the biological findings. Figure 2 brings together the recurring expression patterns and reported tissue organization; neither is intended to convert inferred cell states into validated functional categories. Table 1 summarizes the design and evidentiary limits of representative studies.

## 5. Mechanistic Convergence on Acinar Dysfunction

### 5.1. Disrupted Neural and Neuroendocrine Coordination

Neural dysfunction can reduce secretory stimulation and alter tissue maintenance. Denervation of postganglionic parasympathetic fibers decreases tearing and causes glandular atrophy in mice [2]. Psychological-stress models implicate sympathetic and hypothalamic–pituitary–adrenal signaling in reduced secretion [34]. These distinct settings help separate loss of neural support from excessive inhibitory regulation.

In selected mouse models, noradrenergic signaling suppresses tear secretion through alpha-1A adrenergic receptor (ADRA1A)-dependent regulation of mitochondrial uncoupling protein 2 (UCP2) in acinar and myoepithelial cells [4]. Pharmacological, surgical, chemogenetic, and cell-restricted genetic experiments tested different parts of this pathway. Sympathetic activation suppressed secretion, whereas inhibiting the pathway increased output. These results support a mechanism in the tested models; its contribution to heterogeneous human dry eye remains unresolved.

Corneal nerve injury can also affect epithelial homeostasis. Unilateral nerve severing reduced glandular VIP and was accompanied by bilateral secretory-vesicle abnormalities and iron-associated injury [35]. VIP supplementation or ferrostatin-1 treatment improved tear secretion. These findings connect neural injury with glandular damage, although systemic treatment does not isolate an acinar-cell-specific mechanism.

Chronic psychological stress adds a broader neuroendocrine component through sympathetic and hypothalamic–pituitary–adrenal (HPA) axis activation. In experimental models, reduced stimulated secretion accompanies altered glandular architecture, lipid accumulation, and disturbed immune-cell trafficking. Partial recovery after β-adrenergic blockade supports β-adrenergic involvement in this stress paradigm. Inhibition of glucocorticoid synthesis separately implicates glucocorticoid signaling [34].

These mechanisms should not be conflated. The β-adrenergic and glucocorticoid findings derive from a chronic-stress paradigm, whereas the ADRA1A–UCP2 mechanism was established in other mouse models. Twenty-four-hour profiling additionally revealed disrupted rhythmic immune, metabolic, neuronal, and proliferative outputs despite persistent oscillation of core clock genes [27].

Preserved core clock oscillations alongside altered downstream rhythms suggest disrupted coordination of circadian outputs [27]. The step linking those rhythmic changes to reduced secretion remains unresolved and can be tested with time-resolved functional measurements and targeted interventions [27,28].

Human lacrimal tissue expresses androgen and estrogen receptor transcripts [26]. This finding supports the possibility of direct hormonal responsiveness, but does not measure hormone-induced secretion. The study found no significant sex difference or age correlation in receptor mRNA expression. Cell-specific hormone responses and their relevance to dry eye therefore require separate functional assessment.

Reflex stimulation and direct secretagogue stimulation probe overlapping but different parts of the secretory system [15]. Comparing their responses can help test whether reduced neural drive contributes to hyposecretion. Interpretation still depends on agonist delivery, receptor responsiveness, tissue integrity, and the level at which secretion is measured. Such comparisons do not, by themselves, classify the gland into one of the proposed dysfunction states.

The common functional consequence is a mismatch between the signals reaching the gland and the response the tissue can produce. Receptor-specific experiments can identify selected mechanisms, while tear-output changes alone cannot locate the defect. This distinction guides the choice of neural and epithelial measurements.

### 5.2. Epithelial-Immune Feedback and Persistent Inflammation

Epithelial–immune feedback involves inflammatory signaling within the gland and its effects on secretion. Lacrimal epithelial cells assemble inflammasomes during acute injury and chronic inflammation [36], while interleukin-1 alpha (IL-1α), interleukin-1 beta (IL-1β), and tumor necrosis factor alpha (TNF-α) inhibit neurally evoked protein release in mouse gland lobules [37]. These findings establish components to examine together; the extent of feedback depends on the model.

Inflammasomes provide one experimentally supported link between epithelial stress and inflammatory signaling. Acute-injury and Sjögren-like models show epithelial inflammasome assembly, and chronically inflamed glands show increased IL-1β maturation [36]. Aquaporin 5 (AQP5)-deficient glands and primary epithelial cells exhibit reactive oxygen species (ROS) accumulation, NLR family pyrin domain-containing 3 (NLRP3) activation, caspase-1 and gasdermin D processing, and injury [38]. N-acetyl-L-cysteine reduced pathway activation in cultured cells, while the NLRP3 inhibitor MCC950 attenuated injury and improved tearing in mice. These interventions support ROS–inflammasome involvement in AQP5 deficiency without establishing the same mechanism in all forms of dry eye.

Adaptive immune responses provide another route to epithelial injury. In Aire-deficient mice, clonally expanded CCL5^+^ CD8^+^ memory T cells occur near acini [32]. CD8^+^ T-cell depletion or genetic IFNγ deficiency reduces inflammatory infiltration and acinar atrophy. These interventions support a pathogenic role for these cells and IFNγ in that model. They do not quantify recovery of acinar secretion or establish the dominant lymphocyte mechanism in human Sjögren disease.

Periductal inflammation involves a different spatial setting. In thrombospondin-1-deficient mice, profiling and imaging identify antigen-presenting cells, T follicular helper cells, and germinal-center B cells around ducts [31]. Primary ductal cultures also show inflammatory interleukin-6 (IL-6) secretion and IFNγ-responsive MHC II expression. These observations support epithelial participation in the local response, while the proposed antigen-sampling and recruitment circuit remains a mechanistic hypothesis. Reduced acinar Pigr expression and lower tear secretory IgA provide a separate functional association; their contribution to periductal organization has not been established.

GVHD datasets extend this picture to supporting cells. Fibroblasts show inflammatory expression programs, and endothelial changes involve immune recruitment and cell-junction genes [11]. Stage comparisons also predict fibroblast–T-cell and myeloid–epithelial interactions [29]. These signatures nominate mechanisms involving leukocyte transit, matrix properties, or trophic support; the corresponding tissue activities were not directly measured.

Changes in supporting-cell expression can be informative even when relative abundance changes little [11,29]. Perturbing the implicated population or pathway and measuring the predicted tissue response would test its contribution to persistent inflammation.

These studies support several components of epithelial–immune feedback, with different levels of evidence. Cytokine-induced secretory inhibition and selected lymphocyte mechanisms have experimental support [32,37]. Many stromal, vascular, and myeloid links remain inferred. Whether interrupting one component restores secretion, or whether several components must be targeted together, remains a question for functional studies.

### 5.3. Failure of Secretory Execution and Organelle Homeostasis

Secretory execution depends on membrane transport, vesicle release, and organelle homeostasis. In tear-drainage obstruction models, Rab3D, vesicle-associated membrane protein 8 (VAMP8), and synaptosome-associated protein 23 (SNAP23) expression or localization changes accompany abnormal acinar vesicle fusion [39]. These observations follow a drainage lesion rather than selective perturbation of each protein, and secretion was not uniformly reduced. VAMP8-null mice provide genetic evidence of secretory-granule and protein accumulation in lacrimal acini [40]. The stimulated protein-secretion assay in that study measured salivary output, leaving lacrimal secretion unmeasured. Channel-localization studies inform fluid-transport models, although their contribution to disease requires separate testing [18].

Mitochondrial function links cellular energy supply to secretion. In middle-aged mice, rapamycin improved tearing and mitochondrial and oxidative-stress measures, whereas Mdivi-1 worsened glandular abnormalities [41]. These pharmacological results support mitochondrial involvement but do not selectively establish a causal role for the PTEN-induced kinase 1 (PINK1)–Parkin pathway in acinar cells. Diabetic models provide additional bioenergetic and antioxidant-intervention evidence [42,43]. Some treatments also improve corneal sensitivity and other ocular-surface outcomes, so tear recovery cannot be attributed exclusively to acinar cells [43,44].

Endoplasmic-reticulum stress can disturb the biosynthetic environment required for secretion. AQP5-deficient mice show ER abnormalities, inflammatory changes, and reduced tearing [45]. In an aging model, pharmacological interventions directed at ER stress and protein kinase R-like endoplasmic reticulum kinase (PERK) altered ferroptosis-related injury and glandular function [46]. These findings support model-specific links among proteostasis, injury, and secretion. Although physiological Ca^2+^ release from the ER directly couples stimulation to secretion [16], disease-specific disruption of ER–mitochondrial Ca^2+^ transfer remains a candidate mechanism.

Lipid abnormalities also differ in evidentiary strength. Aging and inflammatory transcriptomes identify altered lipid-related programs [30,36], whereas the aging intervention study measured lipid peroxidation and tested ferroptosis-related pathways [46]. The latter supports a functional contribution in that model; the transcriptomic studies leave lipid flux and its functional effects unresolved.

Autophagy studies provide a complementary genetic test of organelle quality control. Human embryonic stem cells deficient in autophagy-related protein 5 (ATG5) formed lacrimal gland-like organoids with abnormal development, protein aggregation, increased cell death, and reduced secretory-assay activity [47]. These results establish a requirement for autophagy in that developmental culture system. They do not isolate maintenance of mature acinar cells in an intact adult gland. Assessment of pathway flux is also needed because accumulation of an autophagy marker can reflect either increased formation or impaired clearance [41,47].

These findings identify several possible points of secretory failure, including ion transport, vesicle release, energy supply, and proteostasis. A shared hyposecretory phenotype does not distinguish their relative contributions. Measurements should therefore test the component implicated by the intervention.

A further distinction is whether the defect impairs secretion in surviving tissue or causes cell loss, as considered in Section 5.4.

### 5.4. Cell-Fate Consequences of Persistent Stress

Persistent stress can injure secretory tissue as well as suppress its activity. Parasympathetic denervation causes glandular atrophy [2], while aging studies report apoptosis-associated injury alongside mitochondrial abnormalities [41]. These findings should not be treated as the same lesion. The mode of cell death and its contribution to loss of viable acinar tissue require evidence within each model.

AQP5-deficient glands provide evidence for inflammasome-associated pyroptotic injury. ROS accumulation accompanies NLRP3 activation, caspase-1 and gasdermin D processing, and IL-1β release [38]. MCC950 attenuates these changes and improves tearing, while antioxidant treatment reduces pathway activation in primary epithelial cells. The intervention results strengthen attribution to this pathway in AQP5 deficiency.

Ferroptosis-related evidence also extends beyond expression markers. After corneal nerve severing, glands show iron accumulation and altered glutathione peroxidase 4 (GPX4) and transferrin receptor 1 (TfR1), and ferrostatin-1 improves tear secretion [35]. VIP supplementation also attenuates injury and secretory abnormalities. In aging mice, lipid-peroxidation measurements and interventions with ferrostatin-1 or deferoxamine provide additional support for ferroptotic injury [46]. These findings support a contribution in the tested settings, while the proportion of lost cells and cell-specific causal attribution remain incompletely resolved.

Cell-death terminology should follow the mechanism supported by the data. Apoptosis, pyroptosis, ferroptosis, and necroptosis require appropriate molecular and functional evidence; one changed marker is insufficient [48]. Preservation of cell viability helps test the proposed death pathway. Recovery of secretion answers an additional question and should not be required merely to identify a mode of cell death.

Senescence differs from cell death because affected cells remain in the tissue. Aged lacrimal glands show senescence-associated expression programs, p21 expression, and β-galactosidase activity; semaglutide treatment attenuates several of these features [49]. These observations support a senescence-associated phenotype but do not isolate senescence as the sole cause of secretory dysfunction. Identification should combine multiple features and distinguish persistent growth arrest from other non-proliferative states where feasible [50]. The contributions of individual acinar, stromal, and immune populations remain unresolved.

Cell loss reduces the tissue available for secretion, whereas persistent dysfunction can limit the output of cells that remain. Senescence-associated inflammatory signals may further alter the niche [49,50]. Additional workload on surviving acini is a plausible consequence, but has not been established as a compensatory-overload mechanism in these studies. These possibilities motivate the concept of residual functional reserve without providing a validated measure of it.

**Figure 3:**
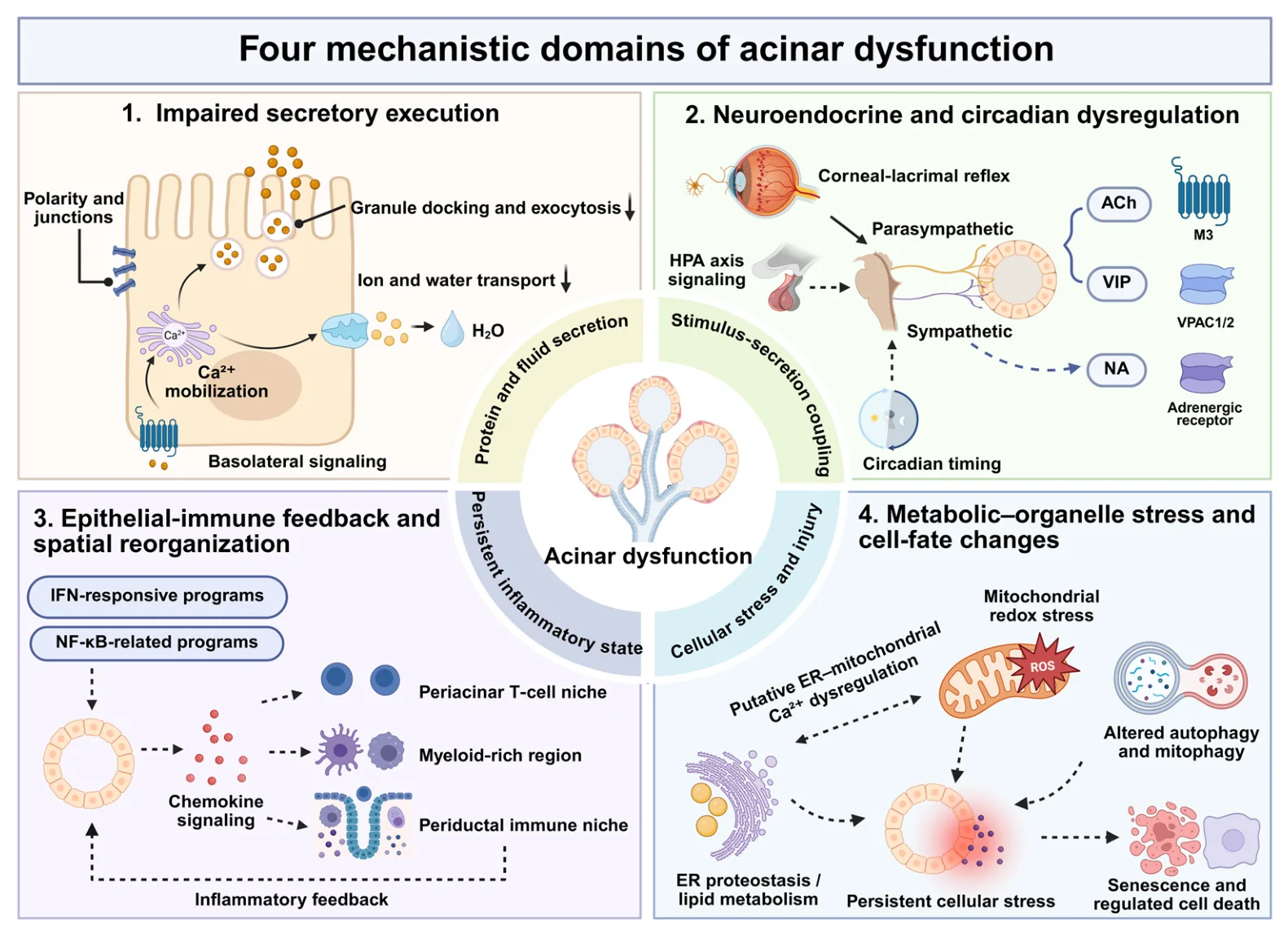
Four mechanistic domains of acinar dysfunction in dry eye disease. The domains summarize evidence from different models and are not successive disease stages. (1) Impaired secretory execution concerns epithelial polarity, Ca^2+^ mobilization, ion and water transport, and granule release. Downward arrows indicate possible functional deficits rather than quantitative effects shared by all models. (2) Neural, neuroendocrine, and circadian inputs influence stimulation and tissue homeostasis. The diagram distinguishes parasympathetic ACh–M3 and VIP–VPAC signaling from context-dependent adrenergic modulation. (3) Epithelial inflammatory programs and immune organization suggest reciprocal interactions. The periacinar, myeloid-rich, and periductal regions combine model-specific findings; the full recruitment and feedback circuit has not been demonstrated in one gland. (4) Mitochondrial stress, ER proteostasis, lipid abnormalities, and autophagy-related changes can accompany injury. The ER–mitochondrial Ca^2+^ link remains putative. Senescence and regulated cell death are distinct possible outcomes, not inevitable consequences of every stress signature. Dashed links denote proposed or context-dependent relationships; solid arrows summarize established physiology or selected experimental findings. Neither line style assigns a formal evidence grade. The figure does not quantify functional reserve or predict reversibility. ACh, acetylcholine; ER, endoplasmic reticulum; HPA, hypothalamic–pituitary–adrenal; IFN, interferon; M3, muscarinic acetylcholine receptor M3; NA, noradrenaline; NF-κB, nuclear factor kappa B; ROS, reactive oxygen species; VIP, vasoactive intestinal peptide; VPAC, vasoactive intestinal peptide receptor; VPAC1/2, vasoactive intestinal peptide receptors 1 and 2.

The available evidence therefore distinguishes injury pathways tested by intervention from cell-state associations and proposed tissue-level consequences. It does not establish a universal progression from stress to irreversible failure. Figure 3 summarizes the mechanistic domains, and Section 5.5 considers what retained tissue and function may mean for recovery.

### 5.5. Residual Functional Reserve and Potential for Recovery

Residual functional reserve is used here to describe the secretory capacity retained by surviving lacrimal tissue and its supporting niche. It includes function that can be recruited by stimulation or recovered by relieving suppression, rather than function requiring replacement of lost tissue. This is a working concept for interpreting recovery potential, not a quantity established by acinar morphology or a single tear measurement. Its interpretation requires attention to viable secretory tissue, epithelial responsiveness, and the neural, ductal, vascular, and stromal conditions under which secretion is assessed.

An experimentally accessible aspect of reserve is the increase in tearing evoked by stimulation. Merino et al. measured basal and corneally stimulated tear secretion in healthy participants and subjects with suspected dry eye or Sjögren’s syndrome [51]. Their measurements illustrate that resting output and the response to stimulation provide different information about tear secretion. However, this response involves the sensory reflex pathway and cannot isolate acinar-cell competence. It also does not establish how much function could recover after treatment of inflammation or tissue injury. We therefore distinguish stimulus-evoked tear reserve from the broader concept of residual functional reserve used in this review.

Mechanistic studies provide complementary evidence for considering functional suppression and structural injury separately. In mice, inhibition of sympathetic signaling increased tear secretion in defined dry eye models [4]. Cytokine exposure also inhibited neurally evoked protein secretion in mouse lacrimal gland lobules [37]. In a rat duct-ligation model, recovery after release differed with obstruction duration, and prolonged obstruction was accompanied by acinar damage and fibrosis [5]. These findings support mechanism-dependent differences in dysfunction and recovery, but they do not validate a common sequence of disease stages.

**Figure 4:**
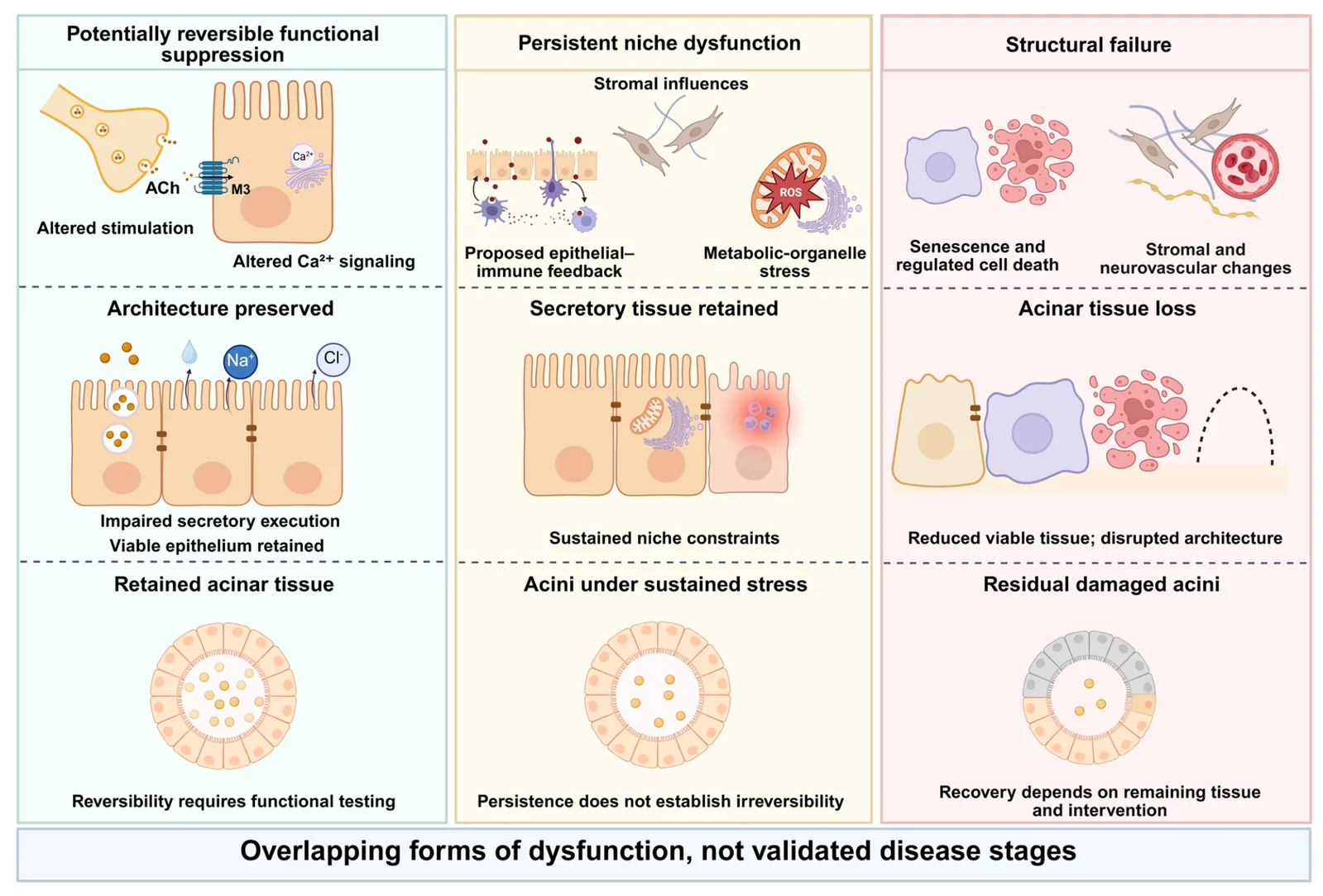
Overlapping forms of lacrimal dysfunction and their implications for functional reserve. The panels illustrate potentially reversible functional suppression, persistent niche dysfunction, and structural failure as proposed descriptions of dominant constraints on secretion. Functional suppression concerns impaired stimulation or secretion in retained epithelium; preserved morphology does not establish reversibility. Persistent niche dysfunction concerns sustained neural, inflammatory, metabolic, or stromal influences on surviving tissue and does not imply permanent loss of function. Structural failure concerns loss of viable acinar tissue or disruption of supporting architecture. The drawings are conceptual examples, not measured states, and do not specify a temporal sequence or a ranking based on tear output. These forms of dysfunction may overlap within a gland. Recovery depends on the remaining tissue, the intervention, and the period of observation, and must be assessed functionally. The three descriptions do not correspond one-to-one to acinar secretory competence, intact-gland output, and lacrimal functional-unit performance. The numbers of granules and molecular symbols are schematic and do not represent quantitative comparisons of secretion or functional reserve. ACh, acetylcholine; M3, muscarinic acetylcholine receptor M3; ROS, reactive oxygen species.

On this basis, we propose three overlapping descriptions of dysfunction (Figure 4). Potentially reversible functional suppression refers to impaired activation or secretory execution in retained epithelium; preserved structure alone does not demonstrate reversibility. Persistent niche dysfunction describes sustained neural, inflammatory, metabolic, or stromal constraints on retained secretory tissue. Structural failure describes loss of viable acinar tissue or disruption of the architecture required for secretion. The distinction between functional suppression and persistent niche dysfunction concerns the dominant constraints and their persistence, rather than a sharp biological boundary.

These descriptions accommodate regional coexistence of functional and structural abnormalities without assigning each gland to a single stage. Spatial studies demonstrate heterogeneous inflammatory and epithelial environments, but have not mapped the three proposed descriptions onto those regions [8,9]. Recovery must be assessed for a defined intervention and observation period; failure to recover within one experiment is insufficient to infer permanent irreversibility.

The three descriptions identify possible forms of dysfunction, whereas the three functional levels identify where an outcome is measured. A given lesion can affect acinar secretory competence, intact-gland secretory output, and lacrimal functional-unit performance to different extents. Evaluating recovery should therefore link the proposed mechanism to the relevant cellular response, glandular secretion, and, when claimed, ocular-surface benefit. Omics and spatial findings can guide these tests but cannot substitute for perturbation or functional measurements. This distinction connects the framework to the validation and restoration strategies discussed in Section 6.

## 6. From Omics Association to Causal Validation and Functional Restoration

### 6.1. Experimental Models and Multicellular Systems

Experimental models should be selected according to the proposed site and mechanism of dysfunction. Pharmacological suppression, neural injury, chronic stress, autoimmune inflammation, aging, diabetes, GVHD, and duct ligation reproduce different combinations of impaired neural input, epithelial stress, inflammation, and structural remodeling [5,9,10,11,30,34,35,42]. Duct ligation is useful for controlled injury–repair studies but does not reproduce the usual pathogenesis of aqueous-deficient dry eye disease. Contralateral controls also require caution after corneal nerve injury because bilateral neuropeptide changes can occur [52].

Comparisons across models can test whether a mechanism recurs under different causes of dysfunction. Age, sex, gland type, disease duration, sampling time, and stimulation conditions influence these comparisons [13]. When the question concerns functional recovery, molecular profiles should be paired with secretion measurements in the same animals or matched experimental cohorts. Ocular-surface outcomes address the additional question of whether glandular improvement benefits the eye.

Organoids allow epithelial mechanisms to be studied under controlled conditions. Adult tissue-derived organoids support genetic perturbation and neurotransmitter-responsive secretion, whereas pluripotent stem cell-derived systems reproduce selected features of lacrimal development and secretory differentiation [12,53,54]. Their cellular composition, maturation, and measured functions differ across culture systems [55]. A positive secretion assay therefore needs to be interpreted alongside the epithelial populations present and the stimulus used.

Multicellular culture can test how selected supporting components affect epithelial function. Lacrimal spheroids combining epithelial, stromal, and endothelial cells showed greater secretory activity, measured by β-hexosaminidase assays, after embedding in a tissue-derived hydrogel [56]. Such approaches complement epithelial organoids by adding defined cellular and matrix interactions relevant to tissue engineering [57]. They still reproduce only part of the neural, vascular, immune, and mechanical environment of an intact gland.

Model choice should follow the question being tested. Organoid secretion provides evidence of epithelial function, while graft survival and tear-protein expression address different aspects of transplantation [12,53,55]. Neither alone establishes regulated tear delivery to the ocular surface. Linking culture experiments to intact-gland measurements is necessary when the claim extends from an epithelial mechanism to restoration of glandular output.

### 6.2. Testing Candidate Mechanisms and Functional Recovery

Testing a candidate mechanism and assessing recovery address related but distinct questions. Mechanistic validation begins with a specific explanation for the observed dysfunction. Cell localization and protein or pathway measurements can establish whether a candidate inferred from omics is present in the relevant tissue context [13,14]. Perturbing the proposed ligand, receptor, or cellular process then tests its contribution to the phenotype. Conditional genetics, localized interventions, and organoid manipulation offer different degrees of control over the affected cell population.

The functional endpoint should reflect the mechanism under study. A proposed defect in stimulus–secretion coupling calls for measurements of receptor responses, Ca^2+^ signaling, ion transport, or protein release. Metabolic or organelle measurements help test the intervening process. Target engagement and complementary loss-of-function, gain-of-function, or rescue experiments strengthen causal interpretation [14]. Which tests are needed depends on whether the claim concerns necessity, sufficiency, or reversibility.

Recovery studies ask an additional question: does correcting the proposed defect restore the function that was lost? Cellular assays address acinar secretory competence, whereas basal and stimulated secretion measurements assess output from the gland. Claims of ocular-surface benefit require corresponding tear-film or ocular-surface outcomes. Persistence after treatment withdrawal is relevant when durable restoration is claimed and when withdrawal is experimentally appropriate. These are distinct questions, not compulsory steps in every mechanistic study.

Timing also determines what an intervention can establish. Preventing dysfunction tests a different question from rescuing function after disease is established. Inducible perturbations can separate initiation from maintenance, while interventions in defined sender or receiver populations can test predicted intercellular signaling [13,14]. Comparisons during treatment and after withdrawal can distinguish ongoing pharmacological support from recovery that persists without it.

Human tissue and culture systems help assess whether findings from animal models apply to human lacrimal biology. Organoids have been established from Sjögren-affected tissue, and mixed cultures have been generated from biopsy-sized specimens from donors without dry eye disease [58,59]. These sources answer different questions. Disease subtype, treatment exposure, donor characteristics, culture composition, and functional readouts therefore need to be reported before a model is described as disease-relevant validation.

A mechanism claim needs evidence linking a defined perturbation to the relevant cellular response; a recovery claim needs improvement in the function being assessed. Evidence at both levels is needed when a study attributes functional recovery to a particular mechanism.

### 6.3. Mechanism-Based Restoration of Acinar Function

The descriptions in Section 5.5 suggest questions for selecting and testing interventions. In retained tissue, studies can ask whether altered neural drive or impaired epithelial responses limit secretion. Persistent inflammatory or metabolic constraints may justify testing interventions directed at the surrounding niche. Where tissue has been lost, repair or replacement becomes a separate objective. These possibilities can overlap within one gland and do not assign a fixed treatment to each proposed form of dysfunction.

Neural interventions show that secretion can improve through changes in regulation. Blocking excessive sympathetic signaling increased tearing in several mouse models [4]. In psychological-stress models, β-adrenergic blockade or inhibition of glucocorticoid synthesis improved secretory function and histological abnormalities [34]. The interventions and disease contexts differ, so their benefits should not be attributed to one common acinar mechanism.

Metabolic interventions offer another way to test recovery in surviving tissue. In middle-aged mice, rapamycin treatment increased tear secretion and reduced oxidative injury, with changes in PINK1–Parkin-associated mitophagy [41]. These findings support a connection between mitochondrial maintenance and glandular function in that model. Because the evidence includes pharmacological manipulation, assigning the benefit specifically to acinar PINK1–Parkin signaling requires more selective tests.

Mesenchymal stromal cells and their extracellular vesicles improved tear production and inflammatory outcomes in Sjögren-like mouse models [60,61]. These findings support investigation of immunomodulation, but they do not establish replacement of lost acinar tissue. A randomized trial tested lacrimal injection of allogeneic adipose-derived mesenchymal stromal cells against a vehicle injection in Sjögren-associated dry eye [62]. Both injection groups improved in symptoms relative to a non-randomized observation group. A tear-film stability advantage over vehicle was reported at four weeks, whereas several other improvements from baseline did not differ significantly between groups. The trial therefore provides early clinical evidence without establishing a cell-specific regenerative mechanism.

A Frizzled-targeting WNT mimetic promoted acinar organoid expansion and improved tissue recovery and tear production after lacrimal duct ligation in mice [63]. The in vivo response also involved injury-associated epithelial populations, and the origin of the recovered acinar cells remained unresolved. This study supports WNT-directed repair after a defined injury; it does not establish efficacy across autoimmune, aging-associated, or fibrotic disease. Further evaluation should distinguish expansion of surviving cells from replacement through other epithelial populations.

Organoid transplantation addresses the possibility of replacing or supplementing depleted epithelium. Reported engraftment, maturation, and tear-protein production support parts of this approach [12,53,55,58]. Restoring glandular output additionally requires effective fluid delivery and integration with the host tissue. Whether a graft responds to physiological stimulation and benefits the ocular surface must therefore be tested directly.

Recovery terminology should identify what the experiment has demonstrated. Improved secretion from surviving cells can support functional rescue; correction of a measured supporting-tissue defect can support niche restoration. Regeneration requires evidence of tissue reconstruction, while restored glandular secretion and ocular-surface benefit remain separate functional outcomes. These processes may occur together, but evidence for one should not be used as a substitute for measuring the others.

## 7. Research Priorities

### 7.1. Human Lacrimal Gland Disease Atlases

The studies reviewed here provide substantially more disease-associated single-cell evidence from mice than from human lacrimal glands [8,9,10,11,12,29,30,31,32]. Human organoid and biopsy-culture studies offer useful access to epithelial biology, but they do not yet constitute a representative atlas of diseased glands [58,59]. A priority is to determine which cellular changes recur in human disease and whether they accompany measurable secretory impairment.

Human disease atlases will need to account for differences among surgical, biopsy, donor, and postmortem specimens. Combining single-cell or single-nucleus profiling with spatial and protein imaging can relate expression patterns to preserved tissue architecture [33,64,65,66]. Functional assays and organoid studies would add complementary information where suitable viable tissue is available. The choice of methods should reflect the specimen and question rather than require every platform for every sample.

Tissue source, ischemic interval, preservation, and processing can affect the profiles obtained and should be documented alongside disease duration and treatment exposure [64,65]. Relevant donor phenotypes include tear output, ocular-surface findings, corneal sensitivity, age, sex, and sampling time [1,27,28]. Paired tear proteomic or metabolomic measurements could then test whether accessible tear markers track glandular changes [67,68,69,70]. Their relationship to tissue pathology and secretion would need direct validation.

Shared metadata and independent donor cohorts would make findings easier to compare and replicate [65]. Tear collection and analysis also require documented procedures when molecular tissue profiles are linked to tear biomarkers [71,72]. Depositing data, images, and analysis workflows would allow researchers to examine whether apparent disease differences persist after accounting for sampling and processing. Together, these steps would establish reproducible disease associations in human tissue and prioritize candidates for the mechanistic tests described in Section 6.2.

### 7.2. Tracking Acinar States During Injury and Recovery

The lacrimal omics studies discussed here often compare one or a few sampled disease stages [8,9,10,11,29,30,31,32]. Such comparisons can identify stage-associated expression patterns without establishing that individual cells pass through those states. Pseudotime can suggest relationships to test, but it does not independently determine the direction, duration, or reversibility of a transition [14,73].

Animal studies can combine repeated secretion measurements with tissue sampling from matched cohorts at defined times during injury and recovery [5]. Lineage tracing and inducible perturbation can address cell origin and mechanism when those questions are central [74]. Sampling should also account for physiological rhythms, which can change after corneal nerve injury [75]. In human studies, longitudinal tear and ocular-surface measurements can be linked to clinically available tissue or cultures without assuming that repeat gland biopsies are feasible [58,59].

A central question is whether an expression-defined acinar state predicts a subsequent change in secretion or response to intervention. Testing this relationship requires alignment of molecular sampling with basal and stimulated functional measurements. Intervention timing and observation after withdrawal should be specified where relevant, alongside circadian phase and other biological conditions that affect secretion [27,28,75].

These designs could test whether the overlapping descriptions in Section 5.5 help explain differences in recovery. They should not assume a progression from functional suppression through niche dysfunction to structural failure. Reserve-related hypotheses could instead ask whether viable tissue, secretory responsiveness, and supporting architecture predict recovery after a defined intervention. The effect of injury duration in duct-ligation studies provides one experimental starting point for that question [5].

### 7.3. Standardized Measures of Secretory Rescue

Comparability requires clear reporting of how secretion was measured. Stimulation, anesthesia, environmental conditions, collection interval, and circadian phase can affect tear output [1,27,28]. Collection and processing methods also influence measurements made from tear samples [71,72,76,77,78]. A change in tear volume therefore needs to be interpreted with its protocol and does not identify which cellular process has recovered.

Outcome selection should follow the study question described in Section 6.2. Clinical benefit and cellular mechanism may require different measures, with prespecified primary outcomes and observation times. A systematic review of dry eye trials found substantial variation in outcome measures and instruments [79]. It supports the need for harmonization, but does not establish the multi-level recovery framework proposed here as an agreed core outcome set.

For animal secretion experiments, we recommend reporting the stimulus and dose, administration route, anesthesia, collection method, collection interval, and sampling conditions. Experimental design, biological replication, randomization, and blinding should be described in accordance with the Animal Research: Reporting In Vivo Experiments (ARRIVE) 2.0 guidelines [80]. Tear composition studies additionally need details of collection devices, storage, sample processing, and normalization, as emphasized by tear-research reporting guidance and methodological studies [71,72,76,77,78,81]. Where possible, related tissue, glandular, and ocular-surface measurements should be linked within the same experimental units.

Reporting should allow readers to determine what improved, under which conditions, and for how long. A shared protocol improves comparability; it does not make transcript abundance, tear volume, and ocular-surface status interchangeable measures. The proposed framework will be useful only if its distinctions can be tested reproducibly across models and human samples.

## 8. Conclusions

Lacrimal hyposecretion reflects both epithelial defects and changes in the neural, immune, vascular, and stromal environment that supports secretion. Single-cell and spatial studies help locate these abnormalities, while perturbation experiments test whether candidate mechanisms contribute to functional loss. Distinguishing acinar secretory competence, intact-gland secretory output, and lacrimal functional-unit performance makes the scope of each finding clearer.

Residual functional reserve offers a working concept for considering secretion that surviving tissue and its supporting niche can still recruit. Potentially reversible functional suppression, persistent niche dysfunction, and structural failure describe overlapping constraints whose relevance must be tested against functional recovery. They are not validated disease stages or a clinical treatment algorithm. The next step is to determine which tissue features and responses to defined interventions predict recovery, and whether these relationships hold in human disease.

## Figures and Tables

**Table 1:** Qualitative evidence map of representative single-cell and spatial studies of the lacrimal gland.

Study	Model or Material	Approach	Key Findings	Interpretive Boundary
Delcroix et al., 2023 [7]	Adult C57BL/6J mouse extraorbital lacrimal glands	Single-cell RNA sequencing with selected histological and lineage-related validation	Resolved epithelial complexity, sex-biased transcriptional programs, and candidate epithelial progenitor populations in the normal adult gland.	Pooling, sex, and batch structure may influence subcluster boundaries; candidate progenitor identity requires functional lineage validation.
Mauduit et al., 2022 [8]	BALB/c and NOD.H2b mouse lacrimal glands	Bulk RNA sequencing combined with 10x Genomics Visium spatial transcriptomics	Associated immune-rich regions with macrophage-related expression and identified spatial differences in epithelial metabolic gene signatures.	Visium spots contain multiple cells; regional co-localization and pathway enrichment do not establish cell-specific causality.
Rattner et al., 2023 [9]	BALB/c controls and MRL/lpr and NOD.H2b Sjögren-like mouse models	Single-cell RNA sequencing; in situ hybridization and immunohistochemistry	Identified model-dependent immune composition and distinct interferon-associated epithelial responses across two autoimmune backgrounds.	The analysis was cross-sectional, and relative cell frequencies are sensitive to tissue dissociation and compositional effects.
Tang et al., 2024 [10]	Scopolamine-treated mice exposed to desiccating stress	Single-nucleus RNA sequencing	Identified reduced mature acinar programs with concurrent epithelial-state and immune-signaling changes in pharmacologically induced dry eye.	Nuclear sequencing underrepresents cytoplasmic transcripts, and altered cell proportions do not by themselves demonstrate absolute cell loss.
He et al., 2024 [11]	Mouse model of lacrimal chronic graft-versus-host disease	Single-cell RNA sequencing with immunofluorescence validation	Revealed multicellular epithelial, immune, stromal, and vascular remodeling and reported an Lrg1-high epithelial state.	A single disease window limits temporal inference; predicted cell–cell communication remains associative.
Bannier-Hélaouët et al., 2021 [12]	Adult human lacrimal gland tissue; human and mouse organoids	Single-cell RNA sequencing; organoid culture and functional perturbation	Defined major human lacrimal epithelial populations and showed that cultured organoids predominantly retain duct-associated programs, although selected organoid preparations remained responsive to secretagogues.	The number of human donors was limited, and organoid swelling does not reproduce the complete multicellular niche or integrated tear delivery.
Jin et al., 2026 [29]	Control, acute, and chronic mouse lacrimal graft-versus-host disease	Single-cell RNA sequencing; pseudotime and ligand–receptor analyses; selected immunofluorescence validation	Reported stage-associated acinar metabolic signatures and predicted stromal–immune and myeloid–epithelial interactions; localized infiltrating plasmacytoid dendritic cells and mast cells.	Expression patterns do not measure metabolic flux or establish adaptive benefit. Pseudotime and immune-cell localization do not validate continuous transitions or ligand–receptor signaling.
Pei et al., 2025 [30]	Young and aged mouse lacrimal glands	Bulk transcriptomics integrated with single-cell RNA sequencing	Associated Plin2 expression with immune and metabolic signatures, including reduced expression in fibroblast and monocyte/macrophage/dendritic-cell populations.	Correlation and feature-selection analyses nominate Plin2 but do not establish its causal contribution to lacrimal dysfunction.
Gupta et al., 2026 [31]	Wild-type and thrombospondin-1-deficient mouse lacrimal glands	GeoMx digital spatial profiling; sequencing data processed using GeoMx NGS Pipeline v2.0; selected immunostaining and tear secretory IgA measurements	Identified epithelial transcriptional signatures and a periductal immune niche. Lower acinar Pigr expression accompanied reduced tear secretory IgA.	Spatial association and a parallel tear endpoint do not establish causal epithelial–immune signaling or a causal link between Pigr expression and tear IgA.
Zhu et al., 2026 [32]	Aire-deficient mouse model of autoimmune dacryoadenitis	Single-cell RNA sequencing; single-cell T-cell receptor sequencing; genetic perturbation	Identified clonal CCL5-positive CD8 memory T cells near acini. CD8 T-cell depletion or IFNγ deficiency reduced inflammation and acinar atrophy.	The mechanism was established in a specific genetic mouse model; relevance to heterogeneous human Sjögren disease requires confirmation.

Note: The studies shown are representative examples selected to illustrate distinct experimental designs and evidence relationships; their inclusion does not imply a comparative ranking of methodological quality. This table is a qualitative evidence map and does not constitute a formal evidence-grading or risk-of-bias assessment. Abbreviations: CCL5, C-C motif chemokine ligand 5; CD8, cluster of differentiation 8; GVHD, graft-versus-host disease; IFNγ, interferon gamma; IgA, immunoglobulin A; RNA, ribonucleic acid; MRL/lpr, MRL/MpJ-Fas^lpr^/J; NOD.H2b, non-obese diabetic H-2b congenic strain.

## Data Availability

No new data were created or analyzed in this study. Data sharing is not applicable to this article.

## Abbreviations

The following abbreviations are used in this manuscript:

ACh	acetylcholine
ADRA1A	alpha-1A adrenergic receptor
AQP4	aquaporin 4
AQP5	aquaporin 5
ARRIVE	Animal Research: Reporting In Vivo Experiments
ATG5	autophagy-related protein 5
cAMP	cyclic adenosine monophosphate
CCL5	C-C motif chemokine ligand 5
CD8	cluster of differentiation 8
CFTR	cystic fibrosis transmembrane conductance regulator
CXCL13	C-X-C motif chemokine ligand 13
DED	dry eye disease
ECM	extracellular matrix
ER	endoplasmic reticulum
GPX4	glutathione peroxidase 4
GTPase	guanosine triphosphatase
GVHD	graft-versus-host disease
HPA	hypothalamic–pituitary–adrenal
IFN	interferon
IFNγ	interferon gamma
IgA	immunoglobulin A
IL-1α	interleukin-1 alpha
IL-1β	interleukin-1 beta
IL-6	interleukin-6
M3	muscarinic acetylcholine receptor M3
MHC II	major histocompatibility complex class II
mRNA	messenger RNA
NA	noradrenaline
Na^+^/K^+^-ATPase	Na^+^/K^+^-dependent adenosine triphosphatase
NF-κB	nuclear factor kappa B
NKCC1	Na^+^/K^+^/2Cl^−^ cotransporter 1
NLRP3	NLR family pyrin domain-containing 3
PERK	protein kinase R-like endoplasmic reticulum kinase
pIgR	polymeric immunoglobulin receptor
PINK1	PTEN-induced kinase 1
PMID	PubMed identifier
RNA	ribonucleic acid
ROS	reactive oxygen species
SNAP23	synaptosome-associated protein 23
SNARE	soluble N-ethylmaleimide-sensitive factor attachment protein receptor
TfR1	transferrin receptor 1
TNF-α	tumor necrosis factor alpha
Tyrobp	TYRO protein tyrosine kinase-binding protein
UCP2	uncoupling protein 2
VAMP8	vesicle-associated membrane protein 8
VIP	vasoactive intestinal peptide
VPAC	vasoactive intestinal peptide receptor
VPAC1/2	vasoactive intestinal peptide receptors 1 and 2
WNT	Wnt family/signaling pathway
